# Beyond *P*-values: the promise of Bayesian interpretation in surgical meta-analysis

**DOI:** 10.1097/JS9.0000000000003158

**Published:** 2025-08-05

**Authors:** Kuan-Yu Tai, Pei-Chun Lai, Yen-Ta Huang

**Affiliations:** a Department of Surgery, National Cheng Kung University Hospital, College of Medicine, National Cheng Kung University, Tainan City, Taiwan; b Education Center, National Cheng Kung University Hospital, College of Medicine, National Cheng Kung University, Tainan City, Taiwan; c Department of Pediatrics, National Cheng Kung University Hospital, College of Medicine, National Cheng Kung University, Tainan City, Taiwan

*Dear Editor,*

We read with great interest the recent systematic review and meta-analysis by Liu *et al* that compared robotic and laparoscopic total gastrectomy for gastric cancer^[1]^. Although the authors conducted a methodologically rigorous analysis that demonstrated superior perioperative outcomes for robotic surgery, their findings regarding long-term oncological outcomes merit further discussion from a statistical interpretation perspective. We prepared this correspondence letter in accordance with the TITAN Guidelines 2025 (Supplemental Digital Content, Supplementary File 1, available at: http://links.lww.com/JS9/E844)^[2]^.

The authors reported no significant differences in the 3-year overall survival (OS) [hazard ratio (HR) = 0.795; 95% confidence interval (CI): 0.564–1.119; *P* = 0.289] and disease-free survival (DFS) (HR = 0.771; 95% CI: 0.476–1.247; *P* = 0.289) between the robotic and laparoscopic approaches. Under the traditional frequentist interpretation, *P*-values >0.05 suggested no evidence of benefits for robotic surgery in terms of oncological outcomes. However, this interpretation may inadvertently obscure clinically meaningful insight.

A recent perspective by Patel and Green eloquently illustrated how rigid adherence to *P*-value thresholds can mask therapeutically beneficial findings^[3]^. Through Bayesian reanalysis of several meta-analyses, they demonstrated that substantial probabilities of clinical benefit often exist, despite failing to achieve conventional statistical significance. We used the R code with the multinma package (settings: random-effects model, prior = normal, iter = 4000, warmup = 2000, chains = 4) to perform Bayesian reanalysis of Liu *et al*’s survival data^[4]^. For OS, the median HR was 0.778, with a 95% credible interval (CrI) of 0.459–1.307. Similar to frequentist confidence intervals, this range appeared to extend into the HR > 1 region, suggesting that robotic surgery might perform worse than laparoscopic surgery. However, the advantage of Bayesian analysis lies in its ability to quantify the probability distribution of the treatment effects. The results demonstrated an 85.9% probability that robotic surgery provides an OS benefit compared with laparoscopic surgery (Fig. 1A). Similarly, for DFS, despite the HR crossing unity (HR = 0.778; 95% CrI: 0.413–1.491), the Bayesian analysis suggested a DFS benefit of 80.4% (Fig. 1B). These probabilistic interpretations represent remarkably high probabilities favoring robotic over laparoscopic approaches, contrasting sharply with the frequentist conclusion of “no difference.” The Bayesian perspective suggests that dismissing the oncological promise of robotic surgery based solely on *P*-values may be overly conservative.

**Figure 1.**
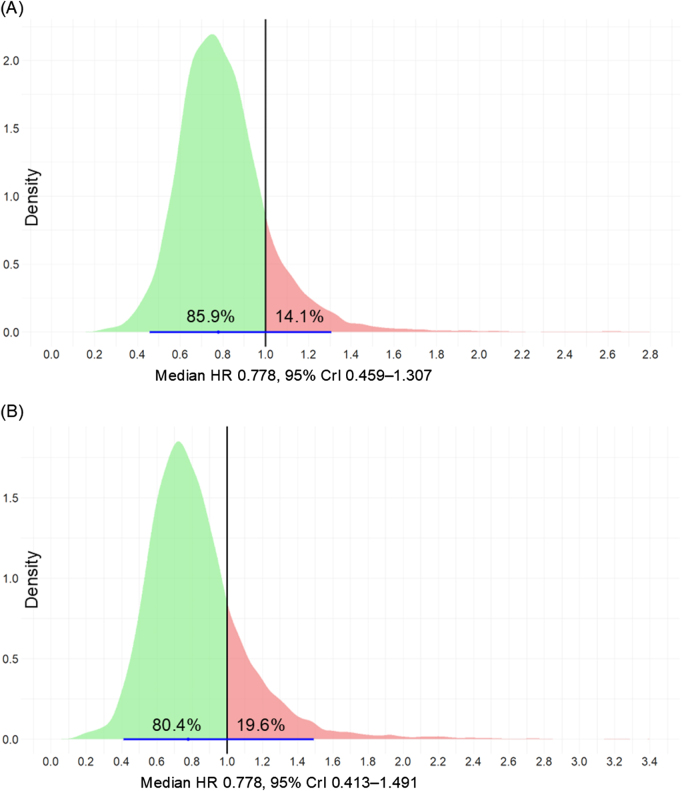
Bayesian reanalysis of survival outcomes for robotic versus laparoscopic total gastrectomy.

Posterior probability distributions of hazard ratios (HRs) for (A) overall survival and (B) disease-free survival. Green areas indicate HR < 1 (favoring robotic surgery); red areas indicate HR > 1 (favoring laparoscopic surgery). Black vertical lines indicate HR = 1 (no difference). Blue dots and lines show the median HR and 95% credible interval (CrIs), respectively.

We commend Liu *et al* for their comprehensive analysis and echo their call that rigorous and larger randomized trials (RCTs) are needed to confirm these potential benefits. However, this is not infeasible, as evidenced by the recent RCT by Feng *et al*, which showed that robotic surgery significantly improved long-term oncological outcomes in patients with middle or low rectal cancer compared to conventional laparoscopic surgery^[5]^. The authors mentioned that their meta-analysis was limited by heterogeneity and bias from non-randomized studies. Alternative methodological approaches, such as target trial emulation using real-world data with propensity score matching and RCT-mimicking design, can help elevate non-RCT studies from association to causal inference and provide complementary evidence while awaiting definitive RCTs^[6]^. This approach has proven valuable in surgical oncology^[7]^, particularly when randomized trials face ethical or practical constraints^[8]^.

In summary, we suggest that future meta-analyses in surgical research should consider reporting both frequentist and Bayesian interpretations^[9]^, as Bayesian methods provide intuitive probability statements about treatment effects, offering a more complete picture of the evidence. These features are particularly valuable in surgical decision-making, where effect sizes with probability estimates, not just statistical significance, drive clinical choice.

## Declaration of Artificial Intelligence use

In accordance with TITAN Guidelines 2025, we declare that generative AI was employed in this manuscript. We have used Claude 4 Opus (Anthropic, released March 2025) exclusively for English language editing after the manuscript was fully drafted. The AI assistance was limited to correcting grammar, improving clarity, and ensuring fluency during sessions conducted between July 8 and 10, 2025. No AI tools were used for study design, data analysis, figure creation, or substantive content generation. The model was operated via Anthropic’s secure cloud web interface with default parameters. Only the authors’ manuscript text was provided to the AI, and no patient data or protected health information was uploaded. All edits suggested by the AI were manually reviewed by the corresponding author and verified by all co-authors for accuracy and scientific integrity. The authors take full responsibility for all content and declare no conflicts of interest with any AI vendors. The single prompt used was: “Please polish the English of the following correspondence letter for submission to the International Journal of Surgery”.

## Data Availability

All data analyzed in this correspondence are publicly available from the published meta-analysis by Liu *et al* (Int J Surg. 2025;doi:10.1097/JS9.0000000000002761). No additional datasets were generated or analyzed during the current study.
